# *hMZF-2*, the Elusive Transcription Factor

**DOI:** 10.3389/fgene.2020.581115

**Published:** 2020-12-18

**Authors:** Alain Chebly, Jean-Marie Peloponese, Evelyne Ségal-Bendirdjian, Jean-Philippe Merlio, Roland Tomb, Edith Chevret

**Affiliations:** ^1^Bordeaux University, INSERM U1053 Bordeaux Research in Translational Oncology (BaRITOn), Cutaneous Lymphoma Oncogenesis Team, Bordeaux, France; ^2^Medical Genetics Unit (UGM), Faculty of Medicine, Saint Joseph University, Beirut, Lebanon; ^3^University of Montpellier, CNRS IRIM-UMR 9004, Research Institute in Infectiology of Montpellier, Montpellier, France; ^4^Université de Paris, INSERM UMR-S 1124, Team: Cellular Homeostasis, Cancer and Therapies, INSERM US36/CNRS UMS 2009, BioMedTech Facilities, Paris, France; ^5^Bordeaux University Hospital Center, Tumor Bank and Tumor Biology Laboratory, Pessac, France; ^6^Department of Dermatology, Faculty of Medicine, Saint Joseph University, Beirut, Lebanon

**Keywords:** telomerase, transcription factor, *hTERT*, Mzf, myeloid zinc finger 1, myeloid zinc finger, MZF-2, myeloid zinc finger 2

## Introduction

The myeloid zinc finger (MZF) protein family encompasses different transcription factors (TFs) including the myeloid zinc finger protein 1 (MZF-1), also known as zinc finger protein 42 (ZNF42) (Hromas et al., 1991). Assessing the role of MZF-1 in the granulocyte colony-stimulating factor (G-CSF)-induced differentiation of neutrophil in mice, Murai et al. (1997) unexpectedly isolated a novel MZF cDNA form that they named MZF-2. They suggested that MZF-1 and MZF-2 are produced from a single gene by using two alternative transcription initiation sites (Murai et al., 1997). The newly MZF-2 isolated was predicted to be longer than MZF-1. In this initial report by Murai et al. (1997) the human and the murine MZF-2 (hMZF-2 and mMZF-2, respectively), were predicted to have a 75.3% identity between their amino acids (aa) sequences. The hMZF-2 and mMZF-2 proteins contain 13 zinc finger motifs each, which are identical to those reported in the MZF-1 protein (Morris et al., 1994; Murai et al., 1997). It was also proposed that both hMZF-1 and hMZF-2 most likely recognize and bind to the same consensus sequences (5′-AGTGGGA-3′ and 5′-CGGGGAGGGGGAA-3′) (Murai et al., 1997). In a complementary study, the same authors investigated only the mMZF-2 form and evaluated its transcriptional regulatory ability in myeloid cells (Murai et al., 1998). In this review, we question the actual existence of hMZF-2 as a transcription factor involved in *hTERT* expression and regulation.

### hMZF-2 and *hTERT* Gene

**Figure 1:**
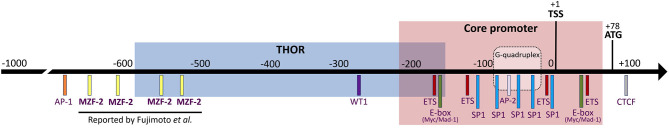
The promoter region of *hTERT* including the core promoter and the TERT hypermethylated oncogenic region (THOR). The transcription start site (TSS, +1) and the translation start site (start codon ATG, +78) are indicated in addition to the binding sites for the “elusive” hMZF-2 (myeloid zinc finger-2) as predicted by Fujimoto et al. (2000) as well as other common transcription factors, such as CTCF (CCCTC-binding factor), ETS (E26 transformation-specific or E-twenty-six), E-box (enhancer-box, where Myc/Mad-family can bind), SP1 (specificity protein 1), AP-2 (activator protein-2), WT1 (Wilms' tumor 1), and AP-1 (activator protein-1). The location of the G-quadruplex structure that can be adopted by the *hTERT* promoter is also represented.

According to the above reports, the hMZF-2 protein was supposed to bind to the distal region of the recently identified telomerase reverse transcriptase (TERT) hypermethylated oncogenic region (THOR) (Figure 1). THOR epigenetic modifications were shown to be a crucial regulator of the *hTERT* gene re-expression in solid tumors and leukemia (Lee et al., 2019) (Figure 1). Indeed, *hTERT* expression, a limiting factor of the telomerase activity (TA), is elevated in 85 to 90% of human cancers, thus promoting survival, proliferation, and invasion capacities of tumor cells (Ramlee et al., 2016). *hTERT* can be regulated through the binding of TFs (either repressors or activators) to its promoter region. MZF-2 was classified among the suppressors of the *hTERT* gene in human and canine (Long et al., 2005; Kyo et al., 2008). Due to the lack of appropriate and validated hMZF-2 antibodies, no chromatin immunoprecipitation experiments were done, and therefore, the binding of MZF-2 to the *hTERT* promoter was reported only as a result of indirect *in vitro* experiments. So far, Fujimoto et al. (2000) predicted that hMZF-2 can bind to 4 sites, all of them being located on the *hTERT* promoter at positions −514, −543, −619, and −687 (Figure 1). Since this initial report, these four binding sites were presented in several figures of book chapters or review articles on telomerase regulation, including recently published ones (Ducrest et al., 2002; Pericuesta et al., 2006; Jafri et al., 2016; Lewis and Tollefsbol, 2016; ElHajj et al., 2017; Heidenreich and Kumar, 2017; Eitsuka et al., 2018; Srinivas et al., 2020), without any additional report that stated unambiguously the existence of *hMZF-2* while the presence of other regulators of the *hTERT* gene, located further upstream of the transcription start site (TSS), were clearly reported to influence the *hTERT* expression, such as the activator protein 1 (AP-1), vitamin D (3) receptor (VDR), signal transducer and activator of transcription 3 (STAT3), and nuclear factor κB (NF-κB) (Ramlee et al., 2016).

### hMZF-2 in the Databases

Blasting the forward and reverse primers (CCGGAGATGGGTCACAGTCC and TTGCTGAACACCTTGCCAC) used by Fujimoto et al. to amplify *MZF-2* transcripts (Fujimoto et al., 2000), we obtained very significant alignments with *MZF-1* and its mRNA variants. Such findings can be explained by the hypothesis that *MZF-2* is transcribed from the same gene as *MZF-1* (Murai et al., 1997). Moreover, the human form hMZF-2 sequence is still absent in the genomic and proteomic databases, while the murine form remains to be validated. In the UCSC Genome Browser on Human (genome.ucsc.edu), the OMIM (omim.org), the NCBI (ncbi.nlm.nih.gov/gene), and the *Ensembl* (ensembl.org) databases, only MZF-1 exists. In the GeneCards database (genecards.org), a search for “*MZF-2*” directs to the *MZF-1* gene and to the biological region LOC110806263 which refers to the TERT 5′ regulatory region on the *hTERT* promoter and citing the paper by Fujimoto et al. (2000). In the proteomic database UniProt (uniprot.org), information concerning MZF-2 in mouse (*Mus musculus*) is available under the label “experimental evidence at transcript level,” but no information is indicated for the human MZF-2 form.

## Discussion

In a recent review article published in 2020, Brix et al. (2020) regrouped information on MZF-1 and its role in regulating cancer invasion. They also discussed *MZF-1* transcript variants. They stated that the first MZF-1 isoform isolated and characterized was believed to be the full-length *MZF-1* (485 aa) until the identification of the long isoforms (734 aa), named *MZF-2a* in mouse and *MZF1B/C* in human (Brix et al., 2020). Brix et al. defined *hMZF-2* as the largest form of *hMZF-1*, or “full-length *hMZF-1*” (Brix et al., 2020). However, the 734 aa full-length *hMZF1* (*MZF1B/1C*) differs in length from the 775 aa *hMZF-2* predicted initially by (Murai et al., 1997; Peterson and Morris, 2000) (Supplementary Figure 1). As for the structural domains in *MZF*, the SCAN domain that mediates interactions between members of a mammalian subfamily of zinc-finger transcription factors is shared between *MZF-1* and *mMZF-2* (uniport.org), while this information is not available fo*r hMZF-2*.

Herein, we summarize the available information regarding *MZF-2* published as original research articles (Murai et al., 1997, 1998; Fujimoto et al., 2000) and those published in review articles (Ducrest et al., 2002; Pericuesta et al., 2006; Jafri et al., 2016; Lewis and Tollefsbol, 2016; ElHajj et al., 2017; Heidenreich and Kumar, 2017; Eitsuka et al., 2018; Srinivas et al., 2020). All the published reports, as well as the search in genomic databases, lead us to be doubtful about the real existence of the human form *hMZF-2*. From these reports, it is not clearly demonstrated whether *hMZF-2* is another isoform of *hMZF-1*. Twenty-three years after its discovery, data concerning hMZF-2 genomic or proteomic sequences are still unpublished. No antibody against the hMZF-2 protein is available. If it is true that hMZF-2 refers to the full-length hMZF-1 as mentioned by Brix DM et al. in 2020, why is this information lacking in the genomic databases? Most of the *hMZF-2* original research articles were published before the availability of a reference genome. However, we aimed to highlight the lack of biological evidence that confirm the existence of *hMZF-2*, functionally differentiate hMZF-2 from hMZF-1, and unequivocally state its ability to regulate the *hTERT* gene. Therefore, we urgently suggest that the four theoretical hMZF-2-binding sites on the *hTERT* promoter should be no longer assigned to this “elusive” transcription factor until further clear experimental evidence is reported (Figure 1). Indeed, the precise identification of the TFs' binding sites on the promoter of the oncogene *hTERT* would refine insights into the epigenetic regulation of *hTERT* activity in cancer.

## Author Contributions

All authors listed have made a substantial, direct and intellectual contribution to the work, and approved it for publication.

## Conflict of Interest

The authors declare that the research was conducted in the absence of any commercial or financial relationships that could be construed as a potential conflict of interest.

## Supplementary Material

Supplementary Figure 1 Pairwise alignment comparison between the human MZF-1 and the mouse MZF-2 proteins showing that the N-terminal region is missing in the full length MZF-1.
