# Supplementary figures and images for: hMZF-2, the Elusive Transcription Factor
Source: Front Genet. 2020 Dec 18;11:581115. doi: 10.3389/fgene.2020.581115 (PMC7793725; doi:10.3389/fgene.2020.581115)

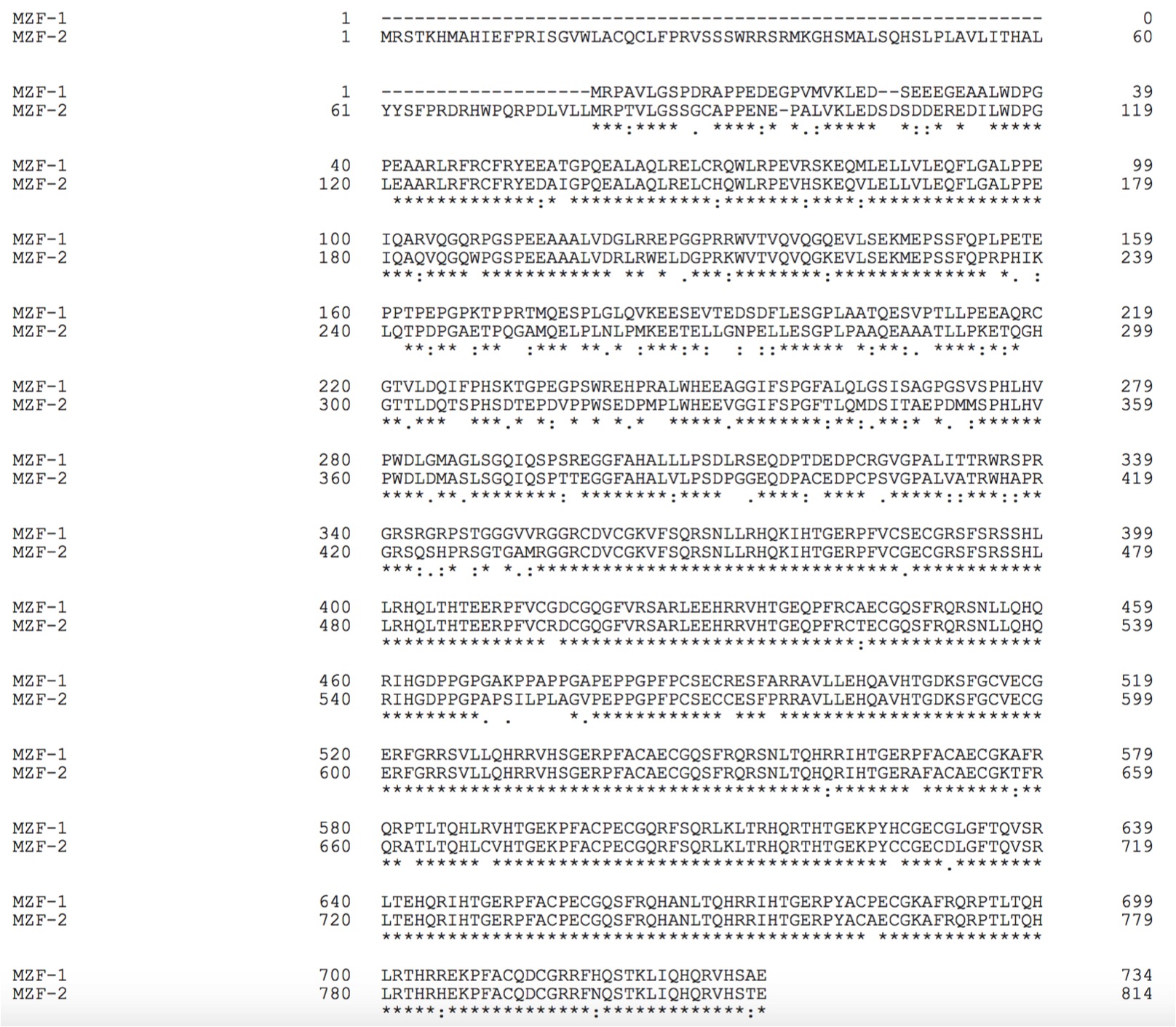

Supplement: Supplementary Figure 1 — Pairwise alignment comparison between the human MZF-1 and the mouse MZF-2 proteins showing that the N-terminal region is missing in the full length MZF-1. [file Image_1.JPEG]
